# Hair chemicals may increase breast cancer risk: A meta-analysis of 210319 subjects from 14 studies

**DOI:** 10.1371/journal.pone.0243792

**Published:** 2021-02-04

**Authors:** Shaohua Xu, Hui Wang, Yeguo Liu, Chengfeng Zhang, Yang Xu, Feng Tian, Lin Mei

**Affiliations:** Department of Oncology, The Second Ward, The PLA Navy Anqing Hospital, Anqing, Anhui Province, China; Nofer Institute of Occupational Medicine, POLAND

## Abstract

**Background:**

The association between personal hair dye use and breast cancer risk is currently debated. The aim of this work is to investigate the association between the use of hair care products and breast cancer risk in women.

**Methods:**

Based on the PRISMA-IPD statement, the PubMed, Embase, Cochrane Library, Web of Science, OVID and Scopus databases were used to identify eligible studies published from inception to 22 April 2020. A pooled odds ratio (OR) with a 95% confidential interval (CI) was calculated to assess this correlation via fixed- or random-effect Mantel-Haenszel models using a heterogeneity Chi^2^ test with a significance level of p<0.1. All statistical tests were performed using StataSE software (version 12.0).

**Results:**

The analyzed data comprised 14 eligible studies with 210319 unique subjects. The pooled results suggested that there was a significant association between the use of hair dyes and breast cancer occurrence (pooled OR = 1.07; 95% CI, 1.01–1.13). Regarding the individual analysis regarding the different types of hair chemicals, permanent hair dye users (pooled OR = 1.08; 95% CI, 1.03–1.14) and rinse users (pooled OR = 1.17; 95% CI, 1.02–1.35) were both found to have a significantly elevated breast cancer risk compared to natural hair subjects, whereas there was an insignificant relationship between the use of semipermanent hair dyes (pooled OR = 1.09; 95% CI, 0.92–1.28) and straighteners (pooled OR = 1.04; 95% CI, 0.96–1.14) and breast cancer risk. No impact on the overall correlation between hair dyes and breast cancer risk due to race (White vs non-White) (pooled OR = 1.05; 95% CI, 0.86–1.29), timing of use (<10 years vs ≥10 years) (pooled OR = 0.96; 95% CI, 0.85–1.08) or dye color (Darker than natural hair vs Lighter than natural hair) (pooled OR = 0.91; 95% CI, 0.62–1.32) was found.

**Conclusions:**

Chemicals in hair dyes may play a role in breast carcinogenesis and increase breast cancer risk.

## Introduction

Worldwide hair dye sales are extraordinarily remarkable, valued at approximately $12 billion per year [1]. Statistically, nearly 1/3 of females in the USA, Europe and East Asia, as well as 5–10% of males in Europe, use some type of hair dye every year, some people use them more than once [2–4]. The excessive application of hair colorants possibly increases users’ risk of cancer and has become a public health concern, as some hair products are potential mutagens and demonstrate endocrine-disrupting characteristics, both of which may be associated with the occurrence of several human carcinomas [5,6]. Hair care products contain more than 5,000 chemicals, of which aromatic amines are mutagenic *in vitro* and carcinogenic in animals and humans [7,8].

The major categories of hair products are temporary (rinse), semipermanent and permanent hair dyes, as well as straightening/relaxing hair chemicals (hereinafter collectively referred to as straighteners); almost all of them are compounded by para-phenylenediamine (PPD: C6H8N2) [9], a powerful skin sensitizer that can induce breast tumors in rats [6]. The potential carcinogenic effect of PPD is presumably attributable to its contamination with 4-aminobiphenyl (4-ABP) during production. In 2010, the International Agency for Research on Cancer (IARC) officially labeled 4-ABP as a human carcinogen because of its mutagenic effect on human DNA [10,11]. Moreover, 4-ABP can permeate the mammary gland to activate estrogen, which is a fundamental etiology of breast cancer [12,13]. Examination of breast ductal epithelial cells shows that 4-ABP-DNA adducts in women who used hair dyes in the past year were 8 times higher than those who never used [14].

A prospective cohort study suggested that hair dyes and straighteners containing endocrine-disrupting chemicals and other carcinogenic compounds might increase the risk of breast cancer, specifically in black women using permanent hair colorants [15]. Breast cancer is still the most common cause of cancer-related death in women, although its overall mortality is gradually decreasing in developed and developing countries [16]. Due to widespread use of hair products, even if they only slightly increase breast cancer risk, they pose tremendous negative consequences for public health. However, previous studies have reached discordant conclusions regarding the impact of personal use of hair chemicals on breast cancer risk by virtue of differences in study design, characteristics of the study population and changes in the chemical formulations of hair products [4,17–24]; the results of three relevant meta-analyses reinforced this contradiction due to differences in included trials and methodology [8,25,26]. To provide instructional evidence-based medical data to inform public health, we undertook a meta-analysis to summarize all the scientific data on the association between hair chemicals and new-onset breast cancer.

## Methods

### Search strategy and study design

In light of the PRISMA-IPD Statement [27], electronic searches were performed using six databases, including PubMed, Embase, Cochrane Library, Web of Science, OVID and Scopus, using the following retrieval strategy: ("Breast Neoplasms"[Mesh] OR (breast cancer) OR (breast tumor) OR (breast tumor)) AND ("Hair Dyes"[Mesh] OR (hair dye) OR straightener). No restrictions were required during the retrieval. Potential citations after 22 April 2020 were not included.

In addition to evaluating the relationship between hair coloring and breast cancer risk, the associations between different types of hair products and breast cancer risk were investigated. Furthermore, the impacts of race (White vs non-White), usage duration (<10 years vs ≥10 years) and hair dye color (lighter than natural hair vs darker than natural hair) on the overall correlation between the use of hair dyes and breast cancer risk were explored.

### Inclusion criteria

- Retrospective or prospective clinical studies published in English;

- Female subjects;
- Studies provided the original odds ratio (OR) and its 95% confidence interval (CI) or the raw data to calculate them;
- The exposure element was the use of any type of hair care product;
- The outcomes were clearly classified into cancer events (cases) and nonevents (controls). Hair care products included semipermanent, permanent hair dyes, rinses and straighteners. Cases referred to women who were healthy initially and developed breast cancer during follow-up.

### Exclusion criteria

- Non-English published articles;
- Article type: review, case report, study protocol or conference paper;
- Other elements that did not meet the inclusion criteria.

Two coauthors (HW and YL) separately screened the titles and abstracts of all citations and removed the unmatched citations. The full texts of the remaining potential studies were further scrutinized, and only satisfactory publications were retained. If there were inconsistencies, they were resolved by the third coauthor (CZ).

### Data extraction

The following information was extracted from the qualified studies by two coauthors (HW and YL) using Excel software, version 2016 (Microsoft Corporation, Redmond, Washington, USA): first author, publication year, study duration, study type, nation of origin, median follow-up, mean age, sample size, categories of hair products, race, dye color, and timing of use. If any variabilities were identified, they were addressed by discussion. We additionally evaluated whether these studies assessed menopausal status, age at first birth, parity, family history of breast cancer, education, history of oral contraceptive use, body mass index, smoking history, number of alcoholic drinks per week and marital status.

### Quality assessment

The Cochrane Collaboration's tool was used to assess the methodological quality of all included studies [28]. Bias was evaluated by two coauthors (HW and YL) independently. Any disagreements were resolved by discussion.

### Statistical analysis

The association between the use of personal hair chemicals and breast cancer risk, and the influence of race, use duration and dye color on this overall association, were presented in the form of a pooled OR that was calculated using the crude ORs with their 95% CIs from all the included studies. The heterogeneity among different trials was evaluated via the heterogeneity Chi^2^ test with a significance level of p<0.1 [29]. When the heterogeneity test was not statistically significant (p≥0.1), a fixed-effect Mantel-Haenszel (MH) model was applied to pool the data; if not, a random-effect MH model was used [29]. Publication bias was investigated using Egger’s test with a significance level of p<0.05 and a Begg’s funnel plot. All statistical tests were analyzed via StataSE software, version 12.0 (StataCorp LP, College Station, TX, USA).

## Results

### Search results

**Fig 1:**
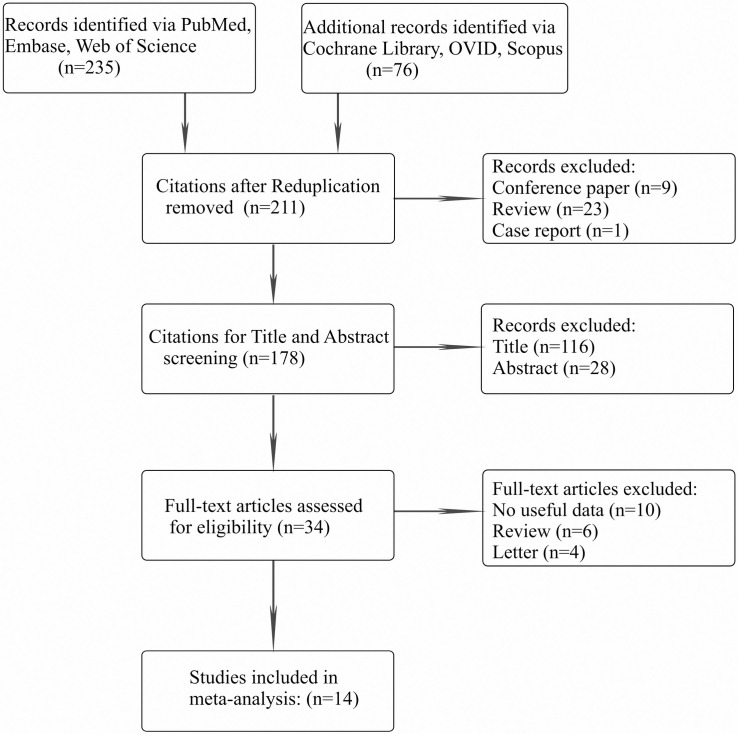
The PRISMA flow diagram of the inclusion process.

We identified a total of 311 potential citations after systematic retrieval, with duplicated studies (n = 100), reviews (n = 23), case reports (n = 1) and conference papers (n = 9) excluded. Of the 178 remaining citations, 144 were removed during title and abstract screening. Therefore, 34 studies were left for full-text scrutiny, and 20 of them were deleted because they lacked useful data (n = 10), were reviews (n = 6) or were letters (n = 4)). Ultimately, 14 eligible publications [4,15,18–24,30–34] with 210319 unique subjects met the inclusion criteria. The PRISMA flow diagram of the inclusion process is shown in **Fig 1.**

### Characteristics of eligible studies

With respect to the included articles, publication years ranged from 1978 to 2019 (median: 1993), median follow-up ranged from 6 to 8.3 years (median: 7.25), and the mean subject age ranged from 48 to 57.5 years old (median: 54.7). The most common article type was a case-control study (78.6%); the most common nation of origin was the USA (64.5%). The most common interview method was a questionnaire survey alone (35.7%) (**Table 1**). As outlined in **Table 2**, seven studies [4,15,18,19,23,31,33] examined semipermanent or permanent hair dyes and rinses, and two [15,24] examined straighteners, while the Green et al. article [20] only examined permanent hair coloring. Several studies specifically considered the impact of race (n = 5), hair dye color (n = 4) and usage duration (n = 8) on the association between hair products and breast cancer risk. **Table 2** also shows other detailed characteristics of the included studies.

**Table 1:** Summary of the subject-number distribution in terms of baseline characteristics.

Characteristic	Studies, No. (%) (N = 14)	Analyzed subjects, No. (%) (N = 210319)
Study type		
Case-control	11 (78.6)	44614 (21.2)
Prospective cohort	3 (21.4)	165785 (78.8)
Publication date, median (range), y	1993 (1978–2019)	
Follow-up, median (range), y*	7.25 (6–8.3)	
Mean age, median (range), y*	54.7 (48–57.5)	
Original nation		
USA	9 (64.5)	62221 (29.6)
China	1 (7.1)	592 (0.3)
Finland	1 (7.1)	27795 (13.2)
Canada	1 (7.1)	255 (0.1)
Iran	1 (7.1)	1052 (0.5)
Australia	1 (7.1)	118404 (56.3)
Interview method		
questionnaire only	5 (35.7)	32581 (15.5)
telephone only	1 (7.1)	1336 (0.6)
Questionnaire and telephone	4 (28.6)	168669 (80.2)
In-person and telephone	1 (7.1)	1804 (0.9)
In-person and questionnaire	2 (14.3)	4877 (2.3)
Hospital files and questionnaire	1 (7.1)	1052 (0.5)
Assessment of menopause		
Yes	7 (50.0)	173549 (82.5)
No	7 (50.0)	36850 (17.5)
Assessment of age at first birth		
Yes	7 (50.0)	174681 (83.1)
No	7 (50.0)	35638 (16.9)
Assessment of parity		
Yes	9 (64.3)	88949 (42.3)
No	5 (35.7)	121370 (57.7)
Assessment of family history of breast cancer		
Yes	6 (42.9)	156574 (74.4)
No	8 (57.1)	53745 (25.6)
Assessment of education		
Yes	11 (78.6)	90634 (43.1)
No	3 (21.4)	119685 (56.9)
Assessment of history of oral contraceptive use		
Yes	8 (57.1)	85715 (40.8)
No	6 (42.9)	124604 (59.2)
Assessment of body mass index		
Yes	8 (57.1)	88627 (42.1)
No	6 (42.9)	121692 (57.9)
Assessment of smoking history		
Yes	10 (71.4)	204094 (97.0)
No	4 (28.6)	6225 (3.0)
Assessment of alcohol drinks per week		
Yes	5 (35.7)	36581 (17.4)
No	9 (64.3)	173738 (82.6)
Assessment of marital status		
Yes	6 (42.9)	34799 (16.5)
No	8 (57.1)	175520 (83.5)

*The median value is calculated using the available data.

**Table 2:** The details of the 14 included studies.

Study (Year)	Study duration	Original nation	Study type	Sample size	Interview method	Hair colorants	Race	Dye color	Dye duration (y)	Ref
Boice (1995)	1926–1982	USA	Case-control	3156	Questionnaire	NA	NA	NA	NA	[34]
Cook (1999)	1983–1990	USA	Case-control	1804	In-person; Telephone	Rinse; Semipermanent HD; Permanent HD; Bleach then dye; Frosting/tipping	White	Lighter; Darker	≤5; 6–15; 16–25; >25	[33]
Dianatinasab (2017)	2014–2016	Iran	Case-control	1052	Hospital files; Questionnaire	NA	NA	NA	NA	[32]
Eberle (2019)	2003–2009	USA	Prospective cohort	46709	Questionnaire; Telephone	Rinse; Semipermanent HD; Permanent HD; Straighteners	Non-Hispanic White; Black; Hispanic White; Other	Lighter; Darker; Combination	<5; ≥5	[15]
Heikkinen (2015)	2000–2007	Finland	Case-control	27795	Questionnaire	Rinse; Semipermanent HD; Permanent HD; Bleach; Partial dye	NA	NA	NA	[4]
Koenig (1991)	1977–1981	USA	Case-control	1336	Telephone	Rinse; Semipermanent HD; Permanent HD	White; non-White	Black; Brown; Red; Blond; Silver/gray	NA	[23]
Mendelsohn (2009)	1996–2000	China	Prospective cohort	592	Questionnaire; In-person	NA	NA	NA	1–2; 3–4; 5–9; ≥10†	[21]
Nasca (1979)	1975–1976	USA	Case-control	349	Questionnaire	Rinse; Semipermanent HD; Permanent HD	NA	NA	NA	[31]
Nasca (1992)	1982–1984	USA	Case-control	3234	Questionnaire; Telephone	Rinse; Semipermanent HD; Permanent HD	White; non-White	NA	<10; 10–19; 19–29; ≥30	[18]
Shore (1978)	1964–1976	USA	Case-control	322	Questionnaire; Telephone	Permanent oxidative HD; Total HD*	NA	NA	1–4; 5–10; ≥11	[30]
Stavraky (1979)	1964–1978	Canada	Case-control	255	Questionnaire	Rinse; Semipermanent HD; Permanent HD	NA	NA	NA	[19]
Wynder (1983)	1979–1981	USA	Case-control	1026	Questionnaire	Permanent HD; Other	Jewish; non-Jewish	NA	<10; 10–19; 19–29; ≥30	[22]
Llanos (2017)	2002–2008	USA	Case-control	4285	Questionnaire; In-person	Total HD; Straighteners	White; Black	Lighter; Darker; Medium	1–10; ≥10	[24]
Green (1987)	1976–1982	Australia	Prospective cohort	118404	Questionnaire; Telephone	Permanent HD	NA	NA	≤5; 6–10; 11–15;16–20;>20†	[20]

Abbreviations: HD, hair dye; NA, not assessed in study.

*Total HD refers to permanent or semipermanent hair dye or rinse.

†Dye duration in these studies is only for cases.

### Hair dyes significantly increased breast cancer risk

**Fig 2:**
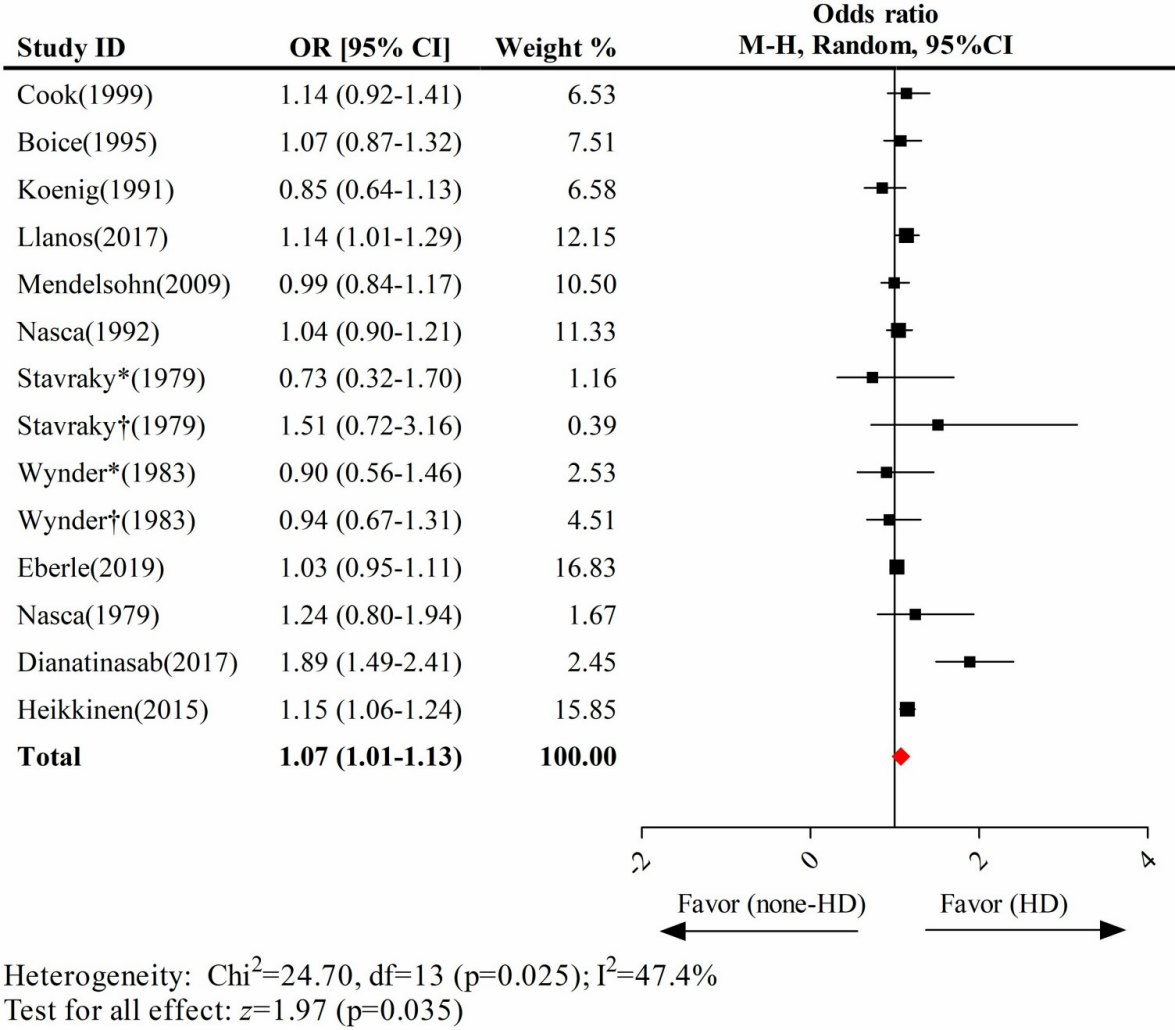
The association between the use of hair dyes and breast cancer risk. The study by Stavraky (1979) contains a Toronto group and a London group; Stavraky* refers to the Toronto group and Stavraky† refers to the London group. The study by Wynder (1983) involves a Jewish group and a non-Jewish group; Wynder* refers to the Jewish group and Wynder† refers to the non-Jewish group. Abbreviations: HD, hair dyes.

For the analysis of the association between hair dyes and breast cancer risk, 12 eligible studies were included. Of these, the study by Stavraky et al. [19] contained subjects in London and Toronto, and the Wynder et al. study [22] reported on Jewish and non-Jewish women, thereby bringing the number of usable studies to 14. The pooled results indicated that the use of hair dyes was significantly associated with breast cancer occurrence (pooled OR = 1.07; 95% CI, 1.01–1.13) (**Fig 2**).

### Permanent hair dyes and rinses were associated with elevated breast cancer risk

**Fig 3:**
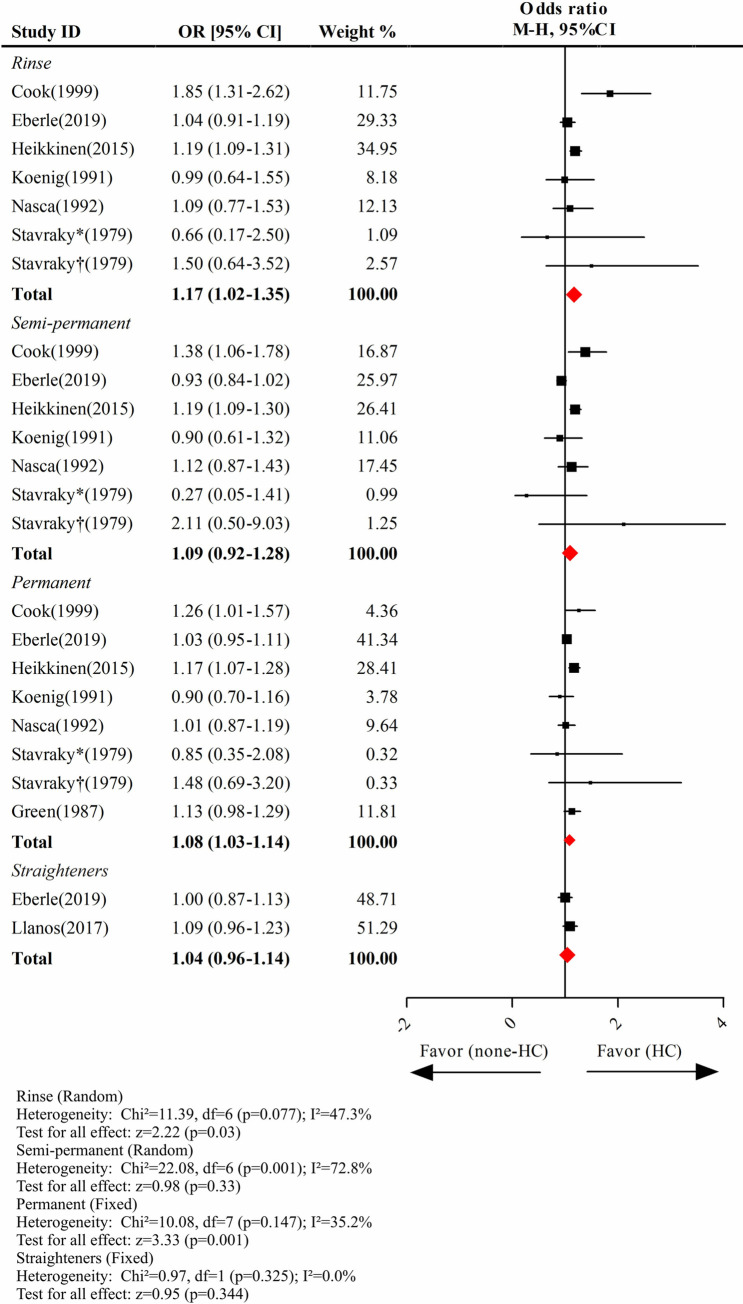
The association between the use of different types of hair chemicals and breast cancer risk. The study by Stavraky (1979) contains a Toronto group and a London group; Stavraky* refers to the Toronto group and Stavraky† refers to the London group. Abbreviations: HC, hair chemicals.

In the investigation of the relationship between different types of hair products and breast cancer risk, the final group of included studies used for the analysis of semipermanent and permanent hair dyes and rinses, as well as straighteners, was not entirely identical. Again, Stavraky et al. [19] classified their subjects into London and Toronto cohorts. The pooled data revealed that breast cancer risk was significantly increased by permanent hair dyes (pooled OR = 1.08; 95% CI, 1.03–1.14) and rinses (pooled OR = 1.17; 95% CI, 1.02–1.35) but was not significantly associated with semipermanent hair dyes (pooled OR = 1.09; 95% CI, 0.92–1.28) or straighteners (pooled OR = 1.04; 95% CI, 0.96–1.14) (**Fig 3**).

### Race, dye color and use duration did not impact the overall relationship between hair dyes and breast cancer risk

**Fig 4:**
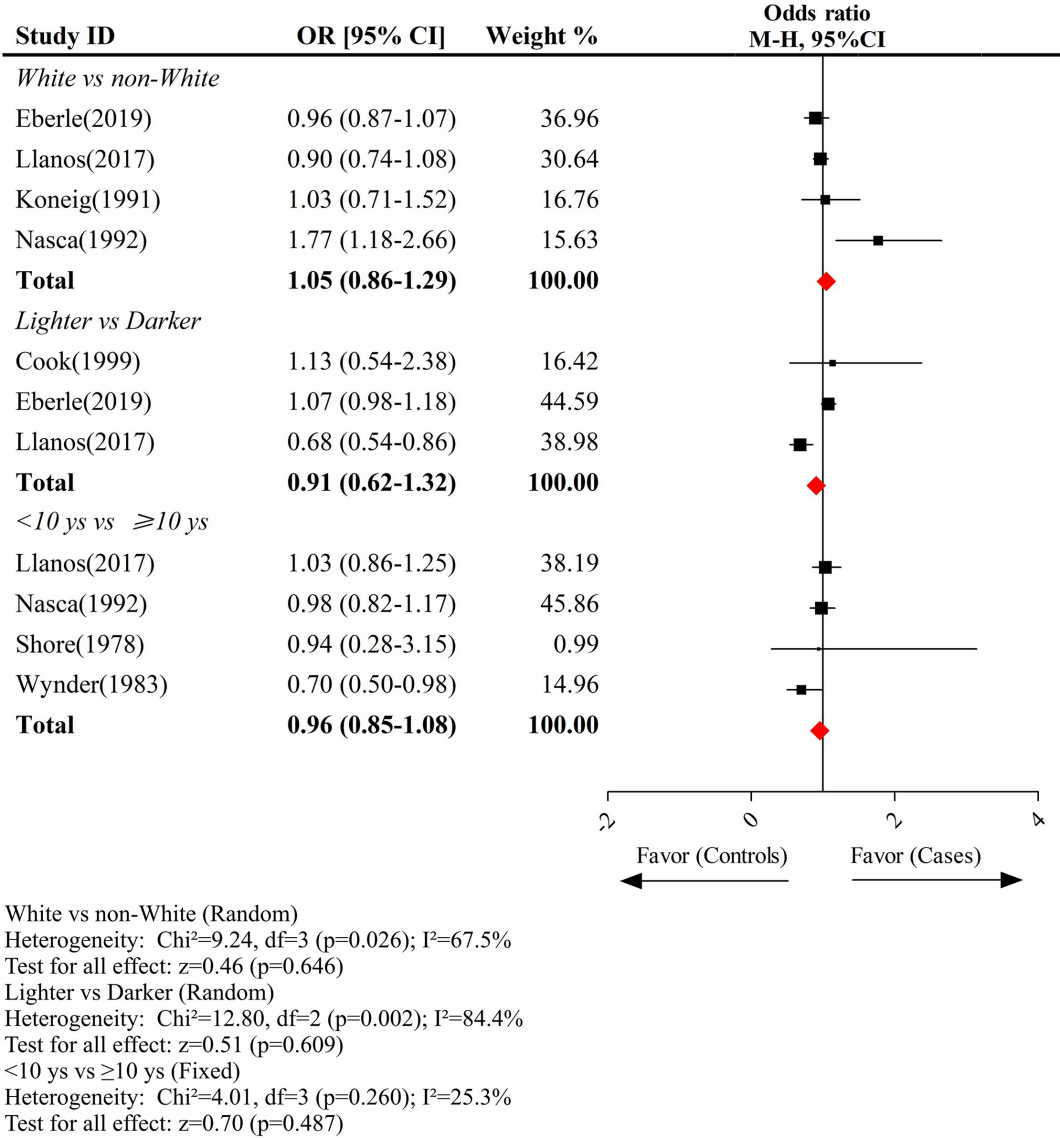
The impact of race, dyeing color and timing of use on the overall association between hair dyes and breast cancer risk.

Although we previously mentioned several articles containing information for this analysis, fewer were usable. As demonstrated in **Fig 4**, the overall difference in breast cancer risk between White females and non-White females after using hair dyes was not significant (pooled OR = 1.05; 95% CI, 0.86–1.29); the results of this analysis mirrored those seen in the comparisons between lighter than natural hair and darker than natural hair (pooled OR = 0.91; 95% CI, 0.62–1.32) and <10 years and ≥10 years (pooled OR = 0.96; 95% CI, 0.85–1.08).

### Risk of bias in included studies

**Fig 5:**
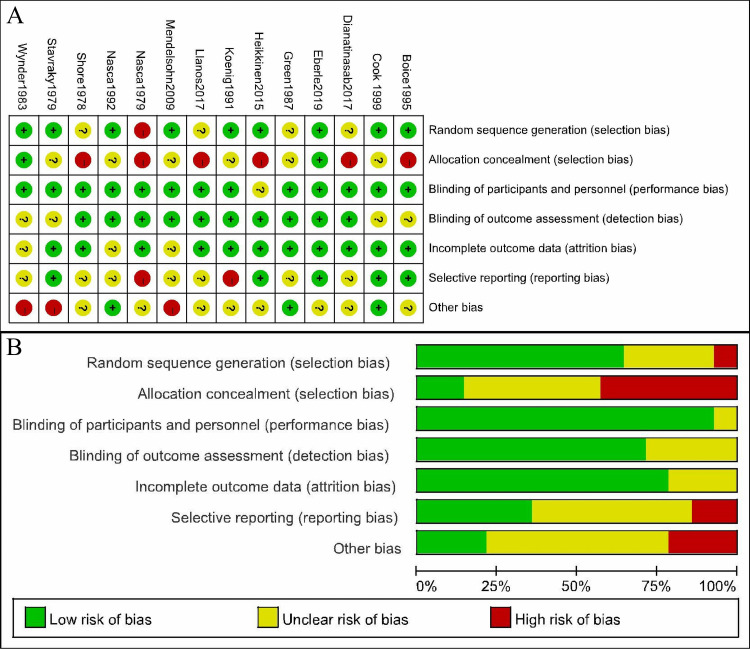
The judgments of the risk of bias summary and the risk of bias graph. A. The risk of bias summary; B. The risk of bias graph.

**Fig 5A** and **5B** present the judgement of the risk of bias summary and the risk of bias graph of the analyzed studies for each “risk of bias” domain, respectively. Nine studies adequately described the method of random sequence generation, while 6 studies did not perform allocation concealment. Thirteen studies had employed some strategies to blind participants and investigators, and 10 studies provided their methods for blinding outcome assessments. There were 11 studies that reported complete outcomes data and 2 studies that showed a high risk of selective reporting. Additionally, 3 studies had an imbalance in baseline characteristics.

### Heterogeneity and publication bias

The following meta-analyses appeared to contain heterogeneity: hair dyes and breast cancer risk, rinse and breast cancer risk, semipermanent hair dye and breast cancer risk, White vs non-White and lighter vs darker; thus, the random-effect MH model was adopted to calculate the value of the pooled OR. The remaining meta-analyses did not manifest any heterogeneity, and their pooled ORs were calculated using the fixed-effect MH model. All publication bias was not significant in terms of the results of Egger’s test (**S1 Table**) or the individual Begg’s funnel plot (**S1–S8 Figs**).

## Discussion

The present large meta-analysis provides robust evidence supporting the conclusion that hair chemicals, particularly in permanent hair colorants and rinses, overtly increase the risk of breast carcinogenesis. Irrespective of our findings demonstrating no impacts of semipermanent hair dyes and straighteners on breast cancer risk, from a public health concern perspective, the results of this study are a meaningful addition to the pool of evidence on the potential association between hair dye use and breast cancer risk, and it further justifies additional research on the topic.

Over the past several decades, many case-control studies and prospective cohort studies have ascribed incredible importance to investigating the relationship between hair care products and new-onset breast cancer [15,17–19,21–23,30,31,33–38]; nevertheless, their conclusions are somewhat controversial. Using scientific methodology, our work concludes that a higher breast cancer risk is found in hair care product users, which is similar to the results of some other research groups [31,33,37]. As previously reviewed, one reason for this positive association is that these hair dyes contain PPD, which is a likely contributor to the carcinogenicity of hair dyes/straighteners. PPD activates the reactive oxygen species (ROS)-mediated mitochondrial pathway and inhibits the NF-κB, mTOR and Wnt pathways that promote healthy cell apoptosis [39]. Furthermore, it damages the lysosomal membrane in fibroblast cells by increasing ROS generation and lipid peroxidation and resulting in the collapse of the mitochondrial membrane potential and cytochrome *c* release [40].

Due to their different chemical compositions, hair colorants are classified as temporary, semipermanent and permanent dyes. A highly sensitive perovskite oxide sensor was used to determine that more than 80% of permanent hair dyes contain PPD [41]. Previous studies have suggested that the use of permanent dyes is closely associated with the occurrence of bladder cancer, lung cancer and Hodgkin's lymphoma [42]. Our study further confirms the positive correlation between the use of permanent dyes and breast cancer risk. By contrast, in 1987, a prospective cohort study found that permanent dyes do not increase the risk of breast cancer, likely due to the particular age of analyzed women (30–55 years) and the limited follow-up duration (only 6 years) [20]. Reaching a conclusion similar to ours, the Sister Study examined a broader age range of subjects and used a longer follow-up period; it observed that personal use of permanent hair dye was associated with a higher breast cancer risk [15].

Rinses, such as fuchsin basic, basic red 2 and Victoria blue B, have gained popularity in the last decade due to their inexpensive formulations, low prices, expedient applications and practical utilization at home. Additionally, study of the association between rinses and cancer risk has advanced recently, and some epidemiological studies have demonstrated insufficient evidence to support the existence of this association [43]. However, one study by Lizier et al. [44] showed that the molecules in hair dyes could be cleaved into noxious PPD. Orange 1 and basic red 51 are commonly applied in rinse formulations; cell experiments demonstrate that they can cause cell necrosis and drive tumorigenesis in healthy cells, respectively [45,46]. One of the predominant targets for the toxicity of most hair dyes is DNA, and many types of rinses can strongly interact with DNA and a result in disturbances in DNA replication, suggesting that rinses pose a potential risk to human health [42]. The findings of our meta-analysis confirm the increased breast cancer risk in rinse users compared with females who have never used them. To date, no previous case-control or prospective cohort studies can provide a direct comparison for ours.

Historically, semipermanent hair dyes have utilized preoxidizing agents deposited on or in the cuticle of the hair shaft as the essential element of their formulations [33]; recently, some oxidants (usually hydrogen peroxide) have been used to manufacture modern semipermanent hair products, similar to permanent hair colorants, that deposit dyes on the cuticle of the hair shaft that then penetrate into the inner portion of the hair shaft [33], resulting in an increased risk to human health. Similarly, sodium hydroxide and thioglycolate, which lack carcinogenic effects in humans, were used as the main active ingredients in traditional straighteners, but the chemical compositions in newer straighteners have changed [47]. Although our work does not uncover positive links between semipermanent hair dyes or straighteners and breast cancer risk, we still recommend avoiding their use, as the publications included in our analysis span a long period, meaning that a combination of traditional and popular semipermanent hair dyes and straighteners were analyzed. Additionally, many current semipermanent hair dyes and straighteners may contain azo bonds that can release aromatic amines after being cleaved [46]. Therefore, future studies focusing on the relationship between modern semipermanent hair dyes and straighteners and breast cancer risk will need to be conducted.

The findings from our study indicate that the overall relationship between hair dyes and breast cancer risk does not vary with race, timing of use and dye color, which is consistent with the results of the Women’s Circle of Health Study (WCHS) [24]. Additionally, of note, the WCHS concludes that breast cancer risk linked to the use of dark hair dye shades in Blacks is higher than in Whites. One possible explanation for this is that patterns of hair dye use vary between Black and White females (the WCHS observed that Black women had a less frequent use of hair dyes but a more frequent use of dark hair dyes than White women). Moreover, the chemical composition of the dark hair dyes used by Whites may be different from those used by Blacks; for instance, the toxicological assessment of some dark hair dyes uncovered higher concentrations of estrogen and endocrine-disrupting compounds in those that were specifically sold to Black women [15], which might correlate with their increased breast cancer risk. Furthermore, dark shades of hair dye contain a large concentration of commercial oxidative agents that pose a greater risk for breast tumorigenesis in Black females [12].

Admittedly, there were some limitations to our work. First, only English publications were included, which might contribute to selection bias and publication bias; indeed, according to the judgment of the risk of bias graph, nearly 50% of included studies were at high risk of selection bias. If we changed the inclusion criteria to decrease the selection bias, it might impact our observed results. Second, since few studies assessed the correlation between the use of straighteners and breast cancer risk, only two qualified articles were included, which might lead to result bias. Third, some studies revealed that, in Black females, straightener users had a greater risk of developing estrogen receptor-negative (ER-) breast cancer than those who never used straighteners [15,24]; because this association was underdocumented and understudied, there was insufficient data for us to analyze. Finally, differences in the chemical formulations of hair care products, dyeing frequency and the breast cancer family history of analyzed subjects across all included studies might cause clinical heterogeneity.

## Conclusion

The use of hair beauty products such as hair dyes, straighteners and rinses may be associated with an increased risk of developing breast cancer in women; therefore, natural hair is preferable to dyed hair with respect to public health. Further studies need to evaluate the relationships between popular semipermanent hair dyes and breast cancer risk and between straighteners and ER- breast cancer risk.

## Supporting information

S1 Checklist PRISMA-IPD checklist of items to include when reporting a systematic review and meta-analysis of individual participant data (IPD). (PDF)

S1 Fig The Begg’s funnel plot with a 95% confidence interval was created to assess the presence of publication bias in hair dye vs never use. (DOCX)

S2 Fig The Begg’s funnel plot with a 95% confidence interval was created to assess the presence of publication bias in rinse analysis. (DOCX)

S3 Fig The Begg’s funnel plot with a 95% confidence interval was created to assess the presence of publication bias in semipermanent hair dye analysis. (DOCX)

S4 Fig The Begg’s funnel plot with a 95% confidence interval was created to assess the presence of publication bias in permanent hair dye analysis. (DOCX)

S5 Fig The Begg’s funnel plot with a 95% confidence interval was created to assess the presence of publication bias in straighteners analysis. (DOCX)

S6 Fig The Begg’s funnel plot with a 95% confidence interval was created to assess the presence of publication bias in White vs non-White analysis. (DOCX)

S7 Fig The Begg’s funnel plot with a 95% confidence interval was created to assess the presence of publication bias in lighter vs darker analysis. (DOCX)

S8 Fig The Begg’s funnel plot with a 95% confidence interval was created to assess the presence of publication bias in <10 years vs ≥10 years analysis. (DOCX)

S1 Table The publication bias by Egger’s test. *Significant level: p<0.05. Abbreviations: ys, years.
(DOCX)

## Abbreviations

PPD: para-phenylenediamine
4-ABP: 4-aminobiphenyl
IARC: International Agency for Research on Cancer
OR: odds ratio
CI: confidence interval
MH: Mantel-Haenszel
ROS: reactive oxygen species
WCHS: Women’s Circle of Health Study
ER-: estrogen receptor-negative
