# Supplementary material for: Hair chemicals may increase breast cancer risk: A meta-analysis of 210319 subjects from 14 studies
Source: PLoS One. 2021 Feb 4;16(2):e0243792. doi: 10.1371/journal.pone.0243792 (PMC7861401; doi:10.1371/journal.pone.0243792)

**S7 Fig. The Begg’s funnel plot with a 95% confidence interval was created to assess the presence of publication bias in Lighter vs Darker analysis.**


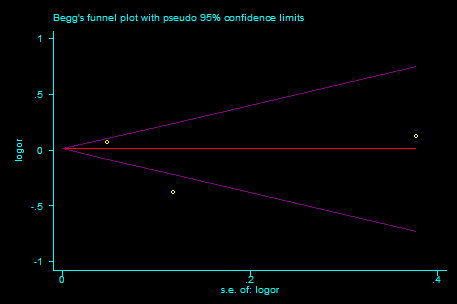

Supplement: S7 Fig — (DOCX) [file pone.0243792.s008.docx]
