# Supplementary material for: Hair chemicals may increase breast cancer risk: A meta-analysis of 210319 subjects from 14 studies
Source: PLoS One. 2021 Feb 4;16(2):e0243792. doi: 10.1371/journal.pone.0243792 (PMC7861401; doi:10.1371/journal.pone.0243792)
Supplement: S1 Table — *Significant level: p<0.05. Abbreviations: ys, years. (DOCX) [file pone.0243792.s010.docx]

| Analyzed label | p value^*^ |
| --- | --- |
| Hair dye vs Never use | 0.638 |
| Hair products use |  |
| Rinse | 0.916 |
| Semipermanent | 0.935 |
| Permanent | 0.936 |
| Straighteners | NA |
| Dye color |  |
| Lighter | 0.341 |
| Darker | 0.691 |
| White vs non-White | 0.359 |
| Lighter vs Darker | 0.682 |
| <10 ys vs ≥10 ys | 0.547 |

**S1 Table. The publication bias by Egger’s test.**

*Significant level: p<0.05.

Abbreviations: ys, years.
